# Computational Efficiency-Based Adaptive Tracking Control for Robotic Manipulators with Unknown Input Bouc–Wen Hysteresis

**DOI:** 10.3390/s19122776

**Published:** 2019-06-20

**Authors:** Kan Xie, Yue Lai, Weijun Li

**Affiliations:** 1 School of Automation, Guangdong University of Technology, Guangzhou 510006, China; kanxiegdut@gmail.com (K.X.); Hot_day@163.com (Y.L.); 2 Guangdong Key Laboratory of IoT Information Technology, Guangzhou 510006, China; 3 Key Laboratory of Ministry of Education, Guangzhou 510006, China; 4 State Key Laboratory of Precision Electronic Manufacturing Technology and Equipment, Guangzhou 510006, China

**Keywords:** sensing and control, computational efficiency, robotic manipulators, hysteresis, adaptive control

## Abstract

In order to maintain robotic manipulators at a high level of performance, their controllers should be able to address nonlinearities in the closed-loop system, such as input nonlinearities. Meanwhile, computational efficiency is also required for real-time implementation. In this paper, an unknown input Bouc–Wen hysteresis control problem is investigated for robotic manipulators using adaptive control and a dynamical gain-based approach. The dynamics of hysteresis are modeled as an additional control unit in the closed-loop system and are integrated with the robotic manipulators. Two adaptive parameters are developed for improving the computational efficiency of the proposed control scheme, based on which the outputs of robotic manipulators are driven to track desired trajectories. Lyapunov theory is adopted to prove the effectiveness of the proposed method. Moreover, the tracking error is improved from ultimately bounded to asymptotic tracking compared to most of the existing results. This is of important significance to improve the control quality of robotic manipulators with unknown input Bouc–Wen hysteresis. Numerical examples including fixed-point and trajectory controls are provided to show the validity of our method.

## 1. Introduction

It is well-known that robotic manipulators are a class of important systems in industrial and academic research [1]. Based on their widespread use in engineering fields, the control of robotic manipulators has attracted much attention of researchers of robotic systems and control science [2,3,4,5,6,7,8]. The modern demand for electronics requires robotic manipulators to be operated in a high-demanding status to reject possible nonlinearities in the closed-loop systems. One of the current research topics is to investigate unknown input nonlinearities in the robotic manipulators.

In practical systems, control inputs are one of the essential units in the closed-loop system and play a key role in maintaining performance and quality [9]. As for the nonlinearities on the input signal, backlash nonlinearity is considered for output feedback control of uncertain nonlinear systems in [10] through backlash inverse. Fu and Xie [11] considered a quantized control problem using a sector bound approach and quantized output feedback systems using a dynamic scaling method [12]. A system with a hysteretic quantizer is considered by Hayakawa et al. [13] to cancel the chattering caused by the logarithmic quantizer. Zhou et al. [14] considered a quantization control problem in a class of systems with parameterized uncertainties and handled it using an adaptive backstepping-based design approach. External disturbances and unknown input nonlinearities are considered for multi-agent systems in [15] and for distributed control of heterogeneous multi-agent systems [16]. Xie et al. [17] addressed unknown input quantization for nonlinear systems and proposed an asymptotic neural-network-based control method. Most recently, faults on control inputs and sensors in multi-agent systems are considered in [18]. Cao et al. [19] used Madelung’s rules to propose a method to model symmetric hysteresis. Furthermore, this method is translated into an algorithm that can be run by digital processors. Hysteresis nonlinearity was considered in [20] for decentralized stabilization of interconnected systems. Later, hysteresis inverse was given in [21] for adaptive output feedback control. It is noted that the parameters of hysteresis in [21] must be available for the control design. The work of [22] considered the tracking control of a magnetic shape memory actuator by combining the modeling technique of an inverse Preisach model and sliding mode control design. The work of [23] studied both time delay and actuator saturation in the formation control of teleoperating systems, which cover robotic systems. The works of [24,25] considered hysteresis nonlinearities in the systems and proposed computational-efficiency-based modeling methods to efficiently and precisely describe hysteresis characteristics.

One has to seek extra controls to handle unknown input coefficients and extra disturbances brought from unknown hysteresis for applications in electronics-based systems. To handle the unknown input coefficients, a Nussbaum function-based control method [26] is considered in the literature [27,28,29,30,31]. The work of [32] used the Nussbaum function for a class of single-input single-output systems. Based on [32], the work of [33] considered unknown control coefficients and model uncertainties and provided a robust control for the segway. Note that single-input single-output systems are not feasible for most of the robotic manipulators, where joint spaces should have six dimensions. Thus, for multiple-input multiple-output systems, some works [34,35,36,37] are provided to handle a group of multiple Nussbaum functions by different control strategies. Most recently, the work of [38] proposed a Nussbaum function with saturated property and used it to address unknown input nonlinearities in robotic systems, with a focus on eliminating the control shock from Nussbaum functions. The work of [39] considered the intrusion detection problem in underwater wireless networks.

Motivated by the above analysis and the technique on the elimination of overparametrization [40], we combine the adaptive control technique and a dynamical gain-based approach to address unknown input Bouc–Wen hysteresis for robotic manipulators. We model the input hysteresis and integrate it with robotic manipulators. Then, two adaptive mechanisms are proposed in our control scheme. Note that computational efficiency is one of the important issues for the implementation of robotic manipulators. We consider such an issue by proposing a control scheme based on two adaptive laws. One adaptive law is used to handle unknown parameter vectors associated with the regression matrix. The other one is used to address input hysteresis and to allow parameters of the hysteresis in each channel of inputs to be different. The two-parameter control scheme plays a key role in improving computational efficiency for potential real-time implementation. A Lyapunov-method-based stability is given to prove the effectiveness of the proposed adaptive scheme. It is shown that even in the presence of unknown input Bouc–Wen hysteresis, the trajectory tracking objective is ensured for robotic manipulators. Moreover, the tracking error is set to be asymptotic within our adaptive control, while most existing results are ultimately bounded. The asymptotic control derived from our method is of importance to high-demanding applications such as manufacturing.

The remaining parts of this paper are organized as follows. We define an unknown input Bouc–Wen hysteresis control problem for robotic manipulators in Section 2. In Section 3, we present a solution containing two adaptive parameters to address the control problem using a dynamical gain-based approach. In Section 4, simulation studies, including fixed-point control and trajectory control, are presented to validate the method’s effectiveness. We summarize the obtained results in Section 5.

## 2. Problem Formulation

A class of robotic manipulators, such as robotic manipulators, are formulated as the following differential equation [1,2,3,4]:

(1) $$D\left(q\right)\ddot{q}+H\left(q,\dot{q}\right)\dot{q}+W\left(q\right)=v,$$

where $q\in R^{L\times 1}$ is a system state vector, $D\left(q\right)\in R^{L\times L}$ denotes an inertial matrix, $H\left(q,\dot{q}\right)\in R^{L\times L}$ represents the Coriolis and centrifugal matrix of the *i*th robotic arm, $W\left(q\right)\in R^{L\times 1}$ denotes the gravitational force vector, $v\in R^{L\times 1}$ means the input of the robotic manipulator and will drive the joint space variable *q* to a predetermined trajectory. Note that the robotic manipulator governed by (1) is capable of modeling jet engines and aircraft.

Here, we specify the input hysteresis nonlinearities as

(2) v=ω(u(t),t),

where

(3) $$\begin{array}{c} \omega \left(u\left(t\right),t\right)=[\omega_{1}\left(u_{1}\left(t\right),t\right),...,\omega_{L}\left(u_{L}\left(t\right),t\right)]. \end{array}$$

**Figure 1:**
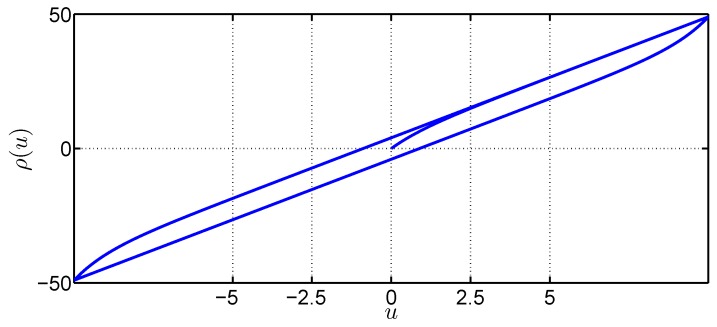
Hysteresis nonlinearities simulated using (4).

Let us consider a single input case for hysteresis nonlinearities modeled as [21,41]

(4) $$\omega \left(u\right)=\mu_{1}u+\mu_{2}\zeta ,$$

where $\mu_{1}$ and $\mu_{2}$ are non-zero constants with $\mu_{1}\mu_{2}>0$, and ζ satisfies

(5) $$\dot{\zeta }=\dot{u}-\varpi |\dot{u}{\left||\zeta \right|}^{n-1}\zeta -\beta \dot{u}{\left|\zeta \right|}^{n}=\dot{u}\psi \left(\zeta ,\dot{u}\right),$$

where n≥1, ϖ>|β| and $\psi \left(\zeta ,\dot{u}\right)=1-\varpi sign\left(\dot{u}\right){\left|\zeta \right|}^{n-1}-\beta {\left|\zeta \right|}^{n}$. From [21,41], one has $\left|\zeta \left(t\right)\right|\leq \sqrt[{n}]{1/(\varpi +\beta )}$. The solution of Equation (4) is depicted in Figure 1, where the hysteresis parameters are set as ϖ=1, n=1, $\mu_{1}=4.5$, β=0, $\mu_{2}=4$, and u=10sin(5t). As shown in Figure 1, the nominal input dynamics preceded by hysteresis phenomena are nonlinear when compared to the linear case wherein v=u.

The control objective in this paper is to construct a two-adaptive-laws-based control scheme for the robotic manipulator (1) with unknown input Bouc–Wen hysteresis (2) so that outputs of the robotic manipulator q(t) track to desired trajectories, that is,

(6) $$\begin{array}{cc} \lim_{t\to \infty } & (q\left(t\right)-q_{d}\left(t\right))=0, \end{array}$$

(7) $$\begin{array}{cc} \lim_{t\to \infty } & (\dot{q}\left(t\right)-{\dot{q}}_{d}\left(t\right))=0. \end{array}$$

**Figure 2:**
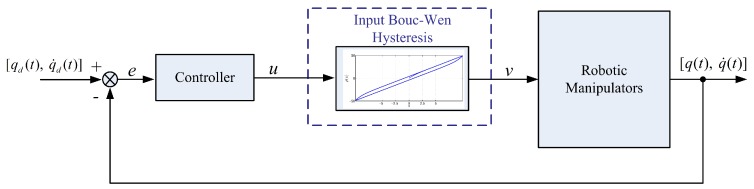
Control diagram for robotic manipulators with unknown input Bouc–Wen hysteresis.

To summarize the design purpose, we give the closed-loop system after applying the control scheme to the robotic manipulators in Figure 2.

## 3. Trajectory Tracking Design for Robotic Manipulators with Unknown Input Hysteresis

In this section, we specify the control method, control design, and the main result for robotic manipulators with unknown input hysteresis. We show that trajectory tracking control is ensured using the proposed adaptive control in the sense of Lyapunov theory.

### 3.1. Control Method

In this subsection, we review the dynamical gain-based approach [42], which will be combined with the adaptive control technique to handle unknown coefficients caused by input hysteresis.

Here, the dynamical gain is given as [42]

(8) $$\begin{array}{cc} N(\chi )= & \;\varpi e^{\chi^{2}}, \end{array}$$

where χ is a real variable. Recalling the result in [42], one has the following result:

**Lemma** **1.** *Let functions V(t) and χ(t) smooth over $\left[0,t_{s}\right)$ with V(t)≥0 and χ(0) bounded. Moreover, χ(t) is a monotonic function. The dynamic loop gain function N is as (8). If one has*

(9) $$\begin{array}{cc} V(t)\leq & \;\beta +\int_{t_{0}}^{t}\dot{\chi }\left(\omega \right)e^{ℑ\omega -ℑt}d\omega \\ & -\int_{t_{0}}^{t}g_{M}N\left(\chi \left(\omega \right)\right)\dot{\chi }\left(\omega \right)e^{ℑ\omega -ℑt}d\omega , \end{array}$$

*where β is a bounded variable and ℑ and $g_{M}$ are positive constants, then the boundedness of V(t) and χ(t) are derived over $\left[0,t_{s}\right)$.*

**Lemma** **2.** *Barbalat’s Lemma [3])**.** Let a function $f\left(t\right)\in C^{1}\left(a,\infty \right)$ and $\lim_{t\to \infty }f\left(t\right)=a$, where a<∞. If $f^{\prime }$ is uniformly continuous, then $\lim_{t\to \infty }f^{\prime }\left(t\right)=0$.*

In what follows, we show how to use dynamical gain (8) to handle unknown input hysteresis in robotic manipulators and how to use one parameter to adaptively tune control coefficients for multiple inputs.

### 3.2. Controller Design

In this subsection, we show the control design to handle input hysteresis in robotic manipulators. Recalling the work in [3], we define

(10) $$\begin{array}{cc} \tilde{q}= & q-q_{d}, \end{array}$$

(11) $$\begin{array}{cc} {\dot{q}}_{r}= & {\dot{q}}_{d}-K_{r}\tilde{q}, \end{array}$$

(12) $$\begin{array}{cc} e= & \dot{q}-{\dot{q}}_{r}, \end{array}$$

where $K_{r}\in R^{L\times L}>0$ is a positive-definite matrix.

**Figure 3:**
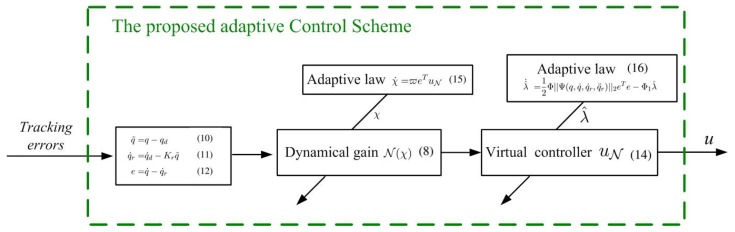
Proposed adaptive control scheme using a dynamical gain-based method.

Now, a controller for robotic manipulators to reject input hysteresis is given as

(13) $$u=N\left(\chi \left(t\right)\right)u_{N},$$

with

(14) $$u_{N}=-\frac{1}{2}{\left||\Psi |\right|}_{2}\hat{\lambda }e-\left(K_{e}+\frac{1}{2}I\right)e,$$

where $\hat{\lambda }$ is an estimate of λ to be detailed later, $K_{e}$ is a positive-definite matrix, and *I* denotes an identity matrix with the dimension of $R^{L\times L}$. The adaptive laws for (13) and (14) are given as

(15) $$\begin{array}{cc} \dot{\chi }= & \varpi e^{T}u_{N}, \end{array}$$

(16) $$\begin{array}{cc} \dot{\hat{\lambda }}\;= & \frac{1}{2}\Phi ||\Psi \left(q,\dot{q},{\dot{q}}_{r},{\ddot{q}}_{r}\right){\left|\right|}_{2}e^{T}e-\Phi_{1}\hat{\lambda }, \end{array}$$

where ϖ, Φ, and $\Phi_{1}$ denote positive constants and $\left|\right|\cdot {\left|\right|}_{2}$ denotes a norm operator. The initial values of λ(t) and χ(t) are set to be non-negative, i.e., λ(0)≥0 and χ(0)≥0. To summarize the design purpose, we show the designed control scheme in Figure 3.

### 3.3. Stability Analysis

Based on the control design in the previous subsection, we use Lyapunov theory to analyze the stability of the proposed adaptive control with a focus on handling unknown input Bouc–Wen hysteresis. Our main result is summarized as follows.

**Theorem** **1.** *Supposing that the robotic manipulators are modeled as (1) with input hysteresis as (4) and (5), the controller is given as (13), and adaptive mechanisms are as (15) and (16), the asymptotic tracking performance in terms of q(t) and $\dot{q}\left(t\right)$ in (1) is guaranteed such that $q\left(t\right)\to q_{d}\left(t\right)$ and $\dot{q}\left(t\right)\to {\dot{q}}_{d}\left(t\right)$ as t→∞.*

**Proof.**  Substituting (13) into (1) leads to

(17) $$\begin{array}{cc} D\left(q\right)\dot{e}+H\left(q,\dot{q}\right)e= & \left[\omega (u,t)-I\right]u_{N}+u_{N}-\Psi \theta , \end{array}$$

where Ψ is a regression matrix and θ is a constant vector with an appropriate dimension. Define a function

(18) $$\begin{array}{c} V=\frac{1}{2}e^{T}D\left(q\right)e+\frac{1}{2}{\tilde{\lambda }}^{2}\Phi^{-1}, \end{array}$$

where $\tilde{\lambda }$ is defined as

(19) $$\begin{array}{c} \tilde{\lambda }=\hat{\lambda }-\lambda , \end{array}$$

with λ being defined later and D(q) being a positive definite matrix following [2]. From (18), one has

(20) $$\begin{array}{cc} \dot{V}=\; & u_{N}+e^{T}\left[\omega \left(u,t\right)-u_{N}\right]+e^{T}\Psi \theta +\dot{\hat{\lambda }}\tilde{\lambda }\Phi^{-1}\\ =\; & u_{N}+e^{T}\left[\omega \left(u,t\right)-u_{N}\right]+\dot{\hat{\lambda }}\tilde{\lambda }\Phi^{-1}+\frac{1}{2}+\frac{1}{2}{\left||\Psi |\right|}_{2}\lambda e^{T}e, \end{array}$$

where $\lambda ={\left||\theta |\right|}_{F}$. Following [2,4], one has that $\dot{D}\left(q\right)-2H\left(q,\dot{q}\right)$ is a skew-symmetric matrix. Substituting (14) and (16) into (20) yields

(21) $$\begin{array}{cc} \dot{V}\leq \; & -e^{T}K_{e}e-\frac{1}{2}e^{T}e+e^{T}\left[\omega \left(u,t\right)-u_{N}\right]+\frac{1}{2}\\ & +\dot{\hat{\lambda }}\tilde{\lambda }\Phi^{-1}-\frac{1}{2}{\left||\Psi |\right|}_{2}e^{T}e\tilde{\lambda }\\ = & -e^{T}K_{e}e-\frac{1}{2}e^{T}e+e^{T}\left[\omega \left(u,t\right)-u_{N}\right]+\frac{1}{2}-\Phi_{1}\hat{\lambda }\tilde{\lambda }\Phi^{-1}, \end{array}$$

where the first inequality is derived after using Young’s inequality. Now, $-\hat{\lambda }\tilde{\lambda }$ in the right-hand side of (21) is changed into

(22) $$\begin{array}{cc} -\hat{\lambda }\tilde{\lambda } & =-\left(\tilde{\lambda }+\lambda \right)\tilde{\lambda }\\ & =-\tilde{\lambda }\tilde{\lambda }+\tilde{\lambda }\lambda \\ & \leq -\tilde{\lambda }\tilde{\lambda }+\frac{1}{2}{\tilde{\lambda }}^{2}+\frac{1}{2}\lambda^{2}\\ & =-(\frac{1}{2}{\tilde{\lambda }}^{2}-\frac{1}{2}\lambda^{2}), \end{array}$$

where both the result in (19) and Young’s inequality are used. From (15) and (22), (21) is further changed into

(23) $$\begin{array}{cc} \dot{V}\leq & -e^{T}K_{e}e-\frac{1}{2}e^{T}e+e^{T}\left[\omega \left(u,t\right)-u_{N}\right]+\frac{1}{2}\\ & -\Phi_{1}\Phi^{-1}\left(\frac{1}{2}{\tilde{\lambda }}^{2}-\frac{1}{2}\lambda^{2}\right)\\ \leq & -e^{T}K_{e}e-\Phi_{1}\Phi^{-1}\frac{1}{2}{\tilde{\lambda }}^{2}+e^{T}[(Diag\left(\mu_{1}\right)N\left(\chi \left(t\right)\right)u_{N}\\ & +Diag\left(\mu_{2}\right)Vec\left(\zeta \right))-u_{N}]+\frac{1}{2}-\frac{1}{2}e^{T}e+\Phi_{1}\Phi^{-1}\frac{1}{2}\lambda^{2}\\ \leq & -e^{T}K_{e}e-\Phi_{1}\Phi^{-1}\frac{1}{2}{\tilde{\lambda }}^{2}+e^{T}\left(N\left(\chi \left(t\right)\right)\mu_{\min}-1\right)u_{N}\\ & +\frac{1}{2}\mu_{\max}+\Phi_{1}\Phi^{-1}\frac{1}{2}\lambda^{2}+\frac{1}{2}\\ \leq & -ℑV\left(t\right)+e^{T}\left(N\left(\chi \left(t\right)\right)\mu_{\min}-1\right)u_{N}+\beta_{0}\\ \leq & -ℑV\left(t\right)+\left(N\left(\chi \left(t\right)\right)\mu_{\min}-1\right)\dot{\chi }\left(t\right)\frac{1}{\varpi }+\beta_{0}, \end{array}$$

where

(24) $$\begin{array}{cc} ℑ= & \min\{\frac{2\delta_{\min}\left\{K_{e}\right\}}{\delta_{\min}\left(D\left(q\right)\right)},\Phi_{1}\}, \end{array}$$

(25) $$\begin{array}{cc} \beta_{0}= & \frac{1}{2}\mu_{\max}+\Phi_{1}\Phi^{-1}\frac{1}{2}\lambda^{2}+\frac{1}{2}, \end{array}$$

(26) $$\begin{array}{cc} Diag(\mu_{1})= & Diag[\mu_{1,1},\mu_{1,2},...,\mu_{1,L}], \end{array}$$

(27) $$\begin{array}{cc} Diag(\mu_{2})= & Diag[\mu_{2,1},\mu_{2,2},...,\mu_{2,L}], \end{array}$$

(28) $$\begin{array}{cc} Vec(\zeta )= & [\zeta_{1},\zeta_{2},\cdots ,\zeta_{L}], \end{array}$$

(29) $$\begin{array}{cc} \mu_{\min}= & \min_{i,j=1,2,\cdots ,L}\left(\mu_{i,j}\right), \end{array}$$

(30) $$\begin{array}{cc} \mu_{\max}= & ||Diag\left(\mu_{2}\right)Vec\left(\zeta \right){\left|\right|}_{2}, \end{array}$$

(31) $$\begin{array}{cc} Vec(\zeta )= & {\left[\zeta_{1},\zeta_{2},\cdots ,\zeta_{L}\right]}^{T}. \end{array}$$

It is clear that *ℑ*, $\mu_{\min}$, and $\beta_{0}$ are positive constants. □

**Remark** **1.** *Here, (23) specifies how to transform the unknown input Bouc–Wen hysteresis control problem into a problem of handling an unknown control coefficient $\mu_{\min}$ and an unknown variable $\beta_{0}$, where $\mu_{\min}$ is given in (29) and $\beta_{0}$ is given in (25). We use the dynamical loop gain (8) and the designed control scheme (14) to make sure that the multiplication in (13) is non-positive. Based on the non-positiveness of $N\left(\chi \left(t\right)\right)u_{N}$, one now finds a upper bound governed by the minimum (29). Please note that even though robotic manipulators are modeled as multiple-input and multiple-output systems, one only needs to handle two scalars, $\mu_{\min}$ and $\beta_{0}$, in (23). This further prompts our adaptive method that uses two adaptive laws to achieve asymptotic control.*

From (23), one has

(32) $$\begin{array}{cc} \;V(t)\leq & \;-V\left(0\right)e^{-ℑt}+\frac{ℑ}{\beta_{0}}+\frac{1}{\varpi }\int_{t_{0}}^{t}\dot{\chi }\left(\omega \right)e^{ℑ\omega -ℑt}d\omega \\ & -\frac{1}{\varpi }\int_{t_{0}}^{t}\mu_{\min}N\left(\chi \left(\omega \right)\right)\dot{\chi }\left(\omega \right)e^{ℑ\omega -ℑt}d\omega \\ ≜ & \;\beta +\frac{1}{\varpi }\int_{t_{0}}^{t}\dot{\chi }\left(\omega \right)e^{ℑ\omega -ℑt}d\omega \\ & -\frac{1}{\varpi }\int_{t_{0}}^{t}\mu_{\min}N\left(\chi \left(\omega \right)\right)\dot{\chi }\left(\omega \right)e^{ℑ\omega -ℑt}d\omega , \end{array}$$

where

(33) $$\begin{array}{c} \beta =-V\left(0\right)e^{-ℑt}+\frac{ℑ}{\beta_{0}}. \end{array}$$

Considering that V(0) is predetermined to be bounded and *ℑ* and $\beta_{0}$ are bounded, one obtains that β is also bounded.

Now, we obtain that (32) is structurally the same as (9). Therefore, the result in *Lemma 1* will hold for (32). That is, from the result in *Lemma 1*, one obtains the boundedness of V(t) and χ(t) [37]. As an immediate result from (14) and (15), one has

(34) $$\begin{array}{cc} \chi (t)-\chi (0) & =\int_{0}^{t}\varpi e^{T}\left(\tau \right)u_{N}\left(\tau \right)d\tau \\ & \geq \int_{0}^{t}\varpi e^{T}\left(\tau \right)e\left(\tau \right)d\tau . \end{array}$$

Note that the boundedness of χ(t) and χ(0) has been ensured and ϖ is a predetermined constant. It is clear that $\int_{0}^{t}\varpi e^{T}\left(\tau \right)e\left(\tau \right)d\tau$ exists and is finite. From *Lemma 2* (*Barbalat’s Lemma*), one has

(35) $$\begin{array}{c} \lim_{t\to \infty }e^{T}e=0, \end{array}$$

so that

(36) $$\begin{array}{c} \lim_{t\to \infty }e\left(t\right)=0. \end{array}$$

Therefore, the convergence in (6) and (7) results. Thus, the proof is completed.

**Remark** **2.** *In Theorem 1, we have proven that even though multiple inputs coexist in the considered robotic manipulators, as shown in (3), the control objective is achieved with computational efficiency using two parameters to be tuned online. In particular, one is in (15) and is responsible for handling unknown input coefficients from hysteresis, and the other one is in (16) and is responsible for addressing the regression matrix from robotic manipulators. This two-parameter adaptive control scheme is feasible due to our unique control design as in (14) and the dynamical gain as in (8). Furthermore, we employ the adaptive control technique to achieve stability for the trajectory tracking control of robotic manipulators with unknown input Bouc–Wen hysteresis.*

## 4. Simulation Example

A two-link articulated robotic manipulator is used for the simulation, which follows the work of [3]. The proposed method is employed to testify to the validity of the proposed control scheme. The manipulator is simulated to move in a horizontal plane and is described as in [3]:

(37) $$\begin{array}{c} \left[\begin{array}{c} D_{11}\;D_{12}\\ D_{21}\;D_{22} \end{array}\right]\left[\begin{array}{c} {\ddot{q}}_{1}\\ {\ddot{q}}_{2} \end{array}\right]+\left[\begin{array}{c} H_{11}\;H_{12}\\ H_{21}\;H_{22} \end{array}\right]\left[\begin{array}{c} {\dot{q}}_{1}\\ {\dot{q}}_{2} \end{array}\right]=\left[\begin{array}{c} v_{1}\\ v_{2} \end{array}\right], \end{array}$$

where

$$D_{11}=\theta_{1}+2\theta_{3}\cos\left(q_{2}\right),$$

$$D_{12}=H_{21}=\theta_{2}+\theta_{3}\cos\left(q_{2}\right)+\theta_{4}\sin\left(q_{2}\right),$$

$$D_{22}=\theta_{2},$$

$$H_{11}=-h{\dot{q}}_{2},\;H_{12}=-h\left({\dot{q}}_{1}+{\dot{q}}_{2}\right),$$

$$H_{21}=h{\dot{q}}_{1},\;H_{22}=0,$$

and

$$h=\theta_{3}\sin\left(q_{2}\right)-\theta_{4}\cos\left(q_{2}\right),$$

with the physical parameters being $\theta ={\left[\theta_{1},\theta_{2},\theta_{3},\theta_{4}\right]}^{T}$. Here, it is noted that the considered system (37) has two inputs and two outputs. As for the system inputs, we specify the input Bouc–Wen hysteresis for each torque as in (4) and (5) with hysteresis parameters $\mu_{1}=4.5,\mu_{2}=4$, ϖ=1, β=0, and n=1. Note that the parameters of hysteresis nonlinearity for each torque can be different according to our result in Section 3. Here, we choose the same hysteresis parameters for simplification. The initial states for the robotic manipulators are also chosen randomly. Note that we need two adaptive laws to implement our method. Here, we set the initials of these two adaptive laws in (15) and (16) as zeroes. That is, $\hat{\lambda }\left(0\right)=0$ and χ(0)=0. Note that the physical parameters to be estimated are vectorized as $\theta ={\left[\theta_{1},\theta_{2},\theta_{3},\theta_{4}\right]}^{T}$. Here, we we use the adaptive law (16) to estimate the scalar $\lambda ={\left||\theta |\right|}_{F}$, not the vector θ. Therefore, the number of estimators drops significantly to only one when compared to the traditional adaptive method. As a result, the computational efficiency is ensured by our method.

In what follows, we give two scenarios that frequently happen in the motion control of robotic manipulators.

### 4.1. Fixed-Point Control Using the Proposed Adaptive Control

**Figure 4:**
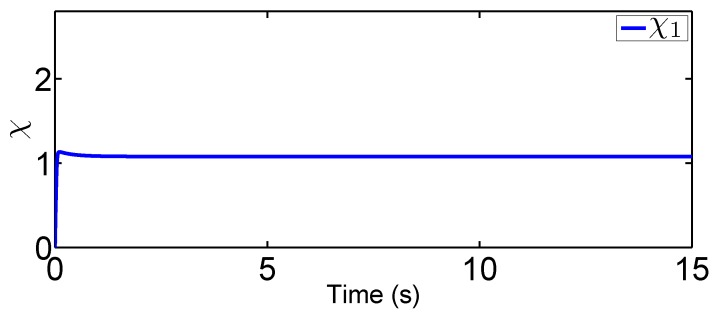
Adaptive law χ for fixed-point control.

**Figure 5:**
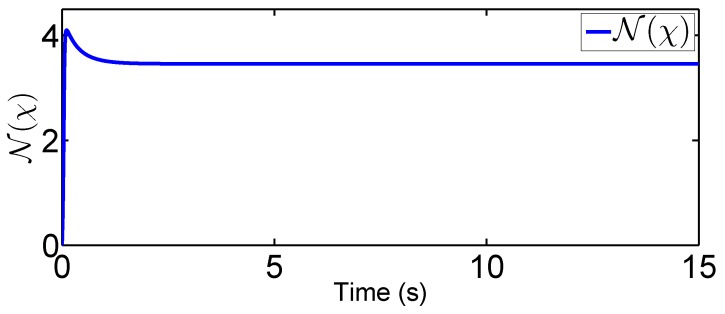
Dynamic loop gain function for fixed-point control.

**Figure 6:**
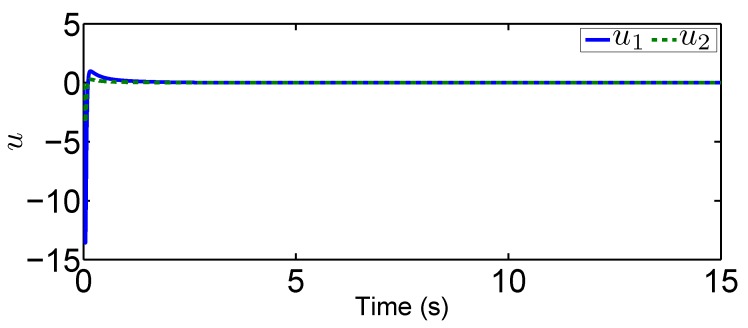
Input signal *u* for fixed-point control.

**Figure 7:**
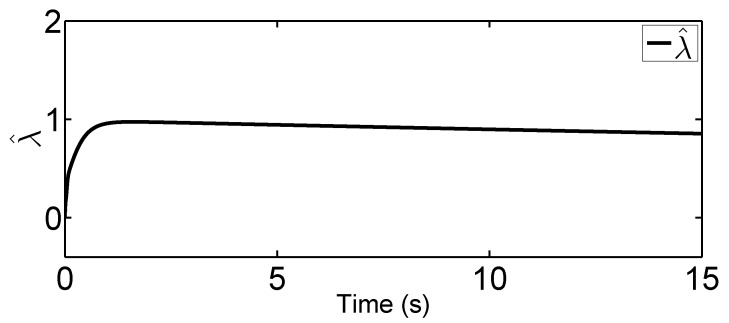
Adaptive law $\hat{\lambda }$ for fixed-point control.

**Figure 8:**
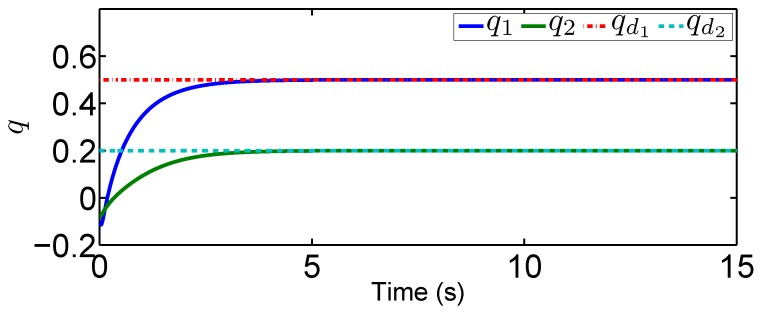
Output *q* for fixed-point control.

**Figure 9:**
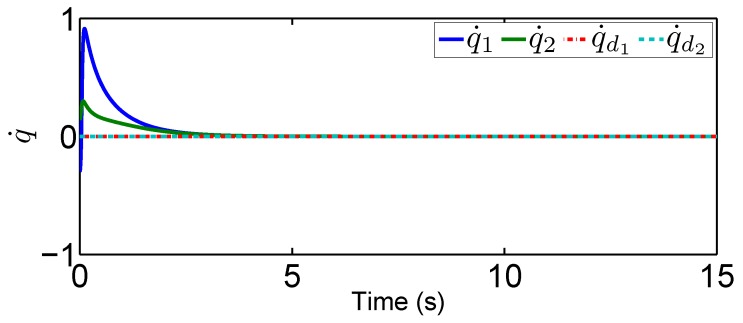
Output $\dot{q}$ for fixed-point control.

In this scenario, we predetermine the desired trajectory as the predetermined points $q_{d}={\left[q_{d1},q_{d2}\right]}^{T}={\left[0.2,0.5\right]}^{T}$ and ${\dot{q}}_{d}={\left[{\dot{q}}_{d1},{\dot{q}}_{d2}\right]}^{T}={\left[0,0\right]}^{T}$. Simulation results are given in Figure 4, Figure 5, Figure 6, Figure 7, Figure 8 and Figure 9. From the observation of Figure 4, Figure 5, Figure 6 and Figure 7, the adaptive variables including χ, N(χ), *u*, and $\hat{\lambda }$ are bounded under unknown input hysteresis and the proposed control method. The outputs of the considered robotic manipulators *q* and $\dot{q}$, as well as the predetermined ones $q_{d}$ and ${\dot{q}}_{d}$, are shown in Figure 8 and Figure 9, where outputs $q_{1}\left(t\right)$ and $q_{2}\left(t\right)$ are, respectively, driven to the predetermined points 0.2 and 0.5 in the presence of the proposed control, while the velocities ${\dot{q}}_{1}\left(t\right)$ and ${\dot{q}}_{2}\left(t\right)$ are regularized to zeroes. Therefore, it is clear that the proposed method is effective in handling input hysteresis in robotic manipulators for the fixed-point control.

### 4.2. Tracking Control Using the Proposed Adaptive Control

**Figure 10:**
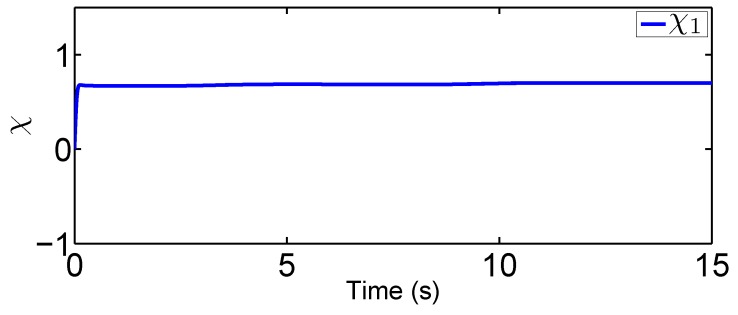
Adaptive law χ for tracking control.

**Figure 11:**
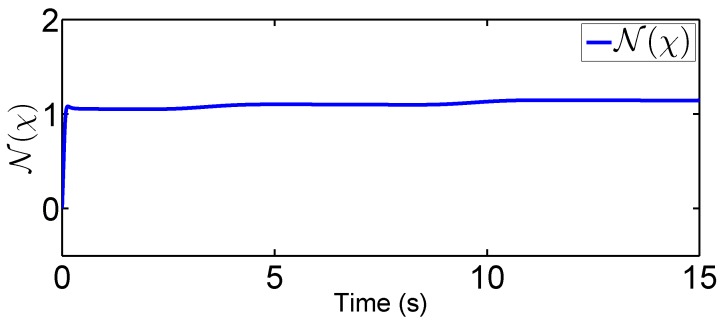
Dynamic loop gain function for tracking control.

**Figure 12:**
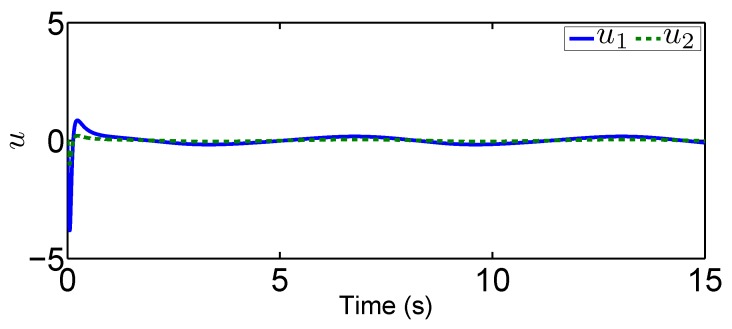
Input signal *u* for tracking control.

**Figure 13:**
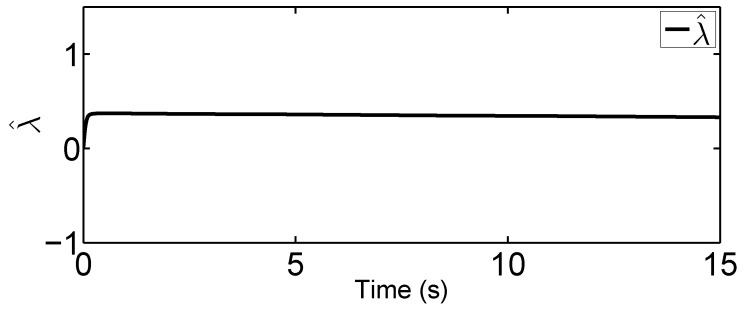
Adaptive law $\hat{\lambda }$ for tracking control.

**Figure 14:**
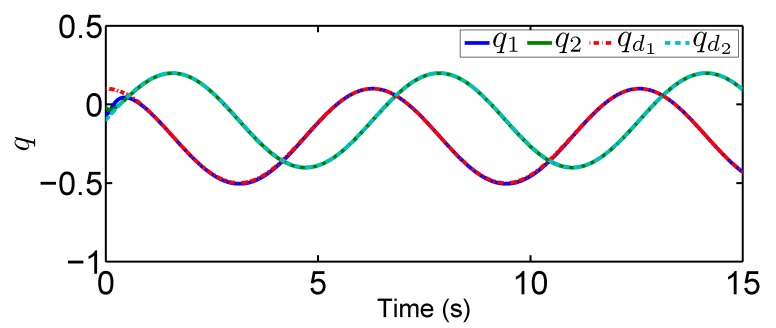
Output *q* for tracking control.

**Figure 15:**
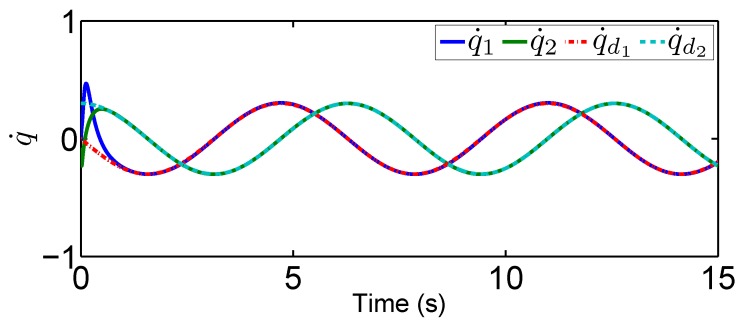
Output $\dot{q}$ for tracking control.

In this tracking control scenario, we set the desired trajectory to be a sine wave. Simulation results for this scenario including the adaptive signals χ, N(χ), $\hat{\lambda }$, and control signal *u*, are given in Figure 10, Figure 11, Figure 12, Figure 13, Figure 14 and Figure 15. In particular, the signal of χ and its dynamical gain N(χ) are given in Figure 10 and Figure 11. The control signal *u* is given in Figure 12. The adaptive law of $\hat{\lambda }$ is given in Figure 13. The results in Figure 10, Figure 11, Figure 12 and Figure 13 show that our method is effective in ensuring all the signals in the closed-loop robotic manipulator are bounded. Finally, the outputs *q* and $\dot{q}$ are provided in Figure 14 and Figure 15, where the proposed method drives the outputs of robotic manipulators to converge to the desired trajectories. This guarantees the effectiveness of the proposed method in achieving tracking control in the presence of unknown input hysteresis.

**Figure 16:**
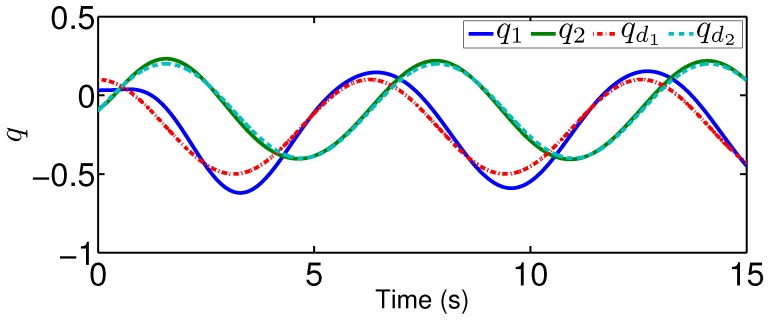
Output *q* for tracking control under the traditional controller.

**Figure 17:**
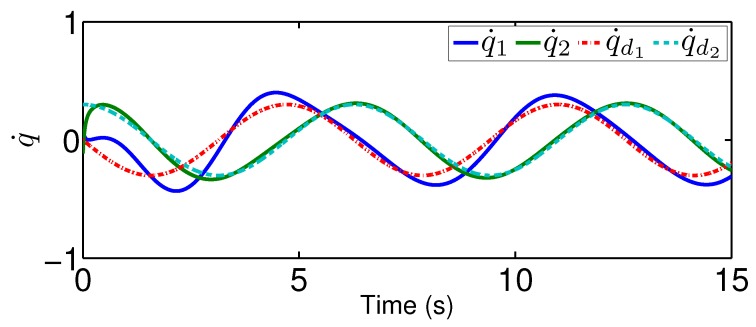
Output $\dot{q}$ for tracking control under the traditional controller.

Moreover, we give the tracking performance under a traditional controller without compensating the hysteresis nonlinearities. For the comparison, we consider the same two-link robotic manipulator as in the previous case. To be specific, a proportional plus derivative controller is applied with $v=-\frac{1}{2}\left(\dot{q}-{\dot{q}}_{d}\right)-\frac{5}{2}\left(q-q_{d}\right)$. The tracking performance in the presence of the traditional controller is given in Figure 16 and Figure 17. From Figure 14, Figure 15, Figure 16 and Figure 17, it is clear that our method provides a better tracking performance compared to the traditional controller.

## 5. Conclusions

In this paper, the problem of input hysteresis is addressed for robotic manipulators. We utilize the adaptive control technique and a dynamical gain-based approach to handle input hysteresis. We use two adaptive parameters to address input hysteresis in robotic manipulators so that computational efficiency is ensured for real-time implementation. Therefore, the proposed adaptive method may be feasible for the purpose of applications. Moreover, we drive the outputs of robotic manipulators to the desired trajectories with zero errors, which guarantees a high level of control quality for robotic manipulators even in presence of unknown input hysteresis. We adopt Lyapunov theory to validate the stability of our method and to prove that all the states and adaptive variables in the closed-loop systems are bounded. In addition, we provide a numerical example including fixed-point and trajectory controls so that the validity of our method is ensured. Future works may extend the proposed method and combine it with advanced learning methods such as those in [43,44,45,46,47,48,49].
